# Cortical Auditory Deafferentation Induces Long-Term Plasticity in the Inferior Colliculus of Adult Rats: Microarray and qPCR Analysis

**DOI:** 10.3389/fncir.2012.00086

**Published:** 2012-11-26

**Authors:** Cheryl Clarkson, M. Javier Herrero-Turrión, Miguel A. Merchán

**Affiliations:** ^1^Instituto de Neurociencias de Castilla y León, Universidad de SalamancaSalamanca, Spain

**Keywords:** brain injury, gene expression profiling, corticofugal projection, long-term post-lesion, adult lesion plasticity

## Abstract

The cortico-collicular pathway is a bilateral excitatory projection from the cortex to the inferior colliculus (IC). It is asymmetric and predominantly ipsilateral. Using microarrays and RT-qPCR we analyzed changes in gene expression in the IC after unilateral lesions of the auditory cortex, comparing the ICs ipsi- and contralateral to the lesioned side. At 15 days after surgery there were mainly changes in gene expression in the IC ipsilateral to the lesion. Regulation primarily involved inflammatory cascade genes, suggesting a direct effect of degeneration rather than a neuronal plastic reorganization. Ninety days after the cortical lesion the ipsilateral IC showed a significant up-regulation of genes involved in apoptosis and axonal regeneration combined with a down-regulation of genes involved in neurotransmission, synaptic growth, and gap junction assembly. In contrast, the contralateral IC at 90 days post-lesion showed an up-regulation in genes primarily related to neurotransmission, cell proliferation, and synaptic growth. There was also a down-regulation in autophagy and neuroprotection genes. These findings suggest that the reorganization in the IC after descending pathway deafferentation is a long-term process involving extensive changes in gene expression regulation. Regulated genes are involved in many different neuronal functions, and the number and gene rearrangement profile seems to depend on the density of loss of the auditory cortical inputs.

## Introduction

The inferior colliculus (IC) is an obligatory relay station for almost all ascending and descending auditory projections and it is a key nucleus for excitatory and inhibitory auditory input convergence (Malmierca and Merchán, 2004). The descending cortico-collicular projection acts as a filter for neuronal responses (Sun et al., 2007). It participates in a positive feedback loop which, in combination with lateral inhibition, sharpens, and adjusts the tuning of neurons in the auditory pathway (Zhang et al., 1997; Jen and Zhang, 1999). The descending auditory corticofugal projection is glutamatergic and therefore excitatory (Feliciano and Potashner, 1995). It is bilateral, but is denser on the ipsilateral side (Saldana et al., 1996; Bajo et al., 2007). Corticofugal deactivation results in unbalanced excitatory/inhibitory IC afferent projections, which induce alterations in the amplitude and latencies of IC neuronal responses (Nwabueze-Ogbo et al., 2002; Popelar et al., 2003), indicating that this projection affects directly the activity of IC neurons.

The developmental auditory system exhibits great ability for network reorganization induced by sensory deprivation (Kandler, 2004; Keuroghlian and Knudsen, 2007). In adults, this capability to induce plastic changes is reduced but still present. In the auditory pathway, plastic changes in the adult have been tested using acoustic stimulation (Norena and Eggermont, 2006), training-behavioral methods (Edeline and Weinberger, 1991a,b, 1992; Gao and Suga, 1998, 2000; Ma and Suga, 2005; Rutkowski and Weinberger, 2005), adaptation after sensory deafferentation (Holt et al., 2005, 2006; Illing et al., 2005; Illing and Reisch, 2006; Rubio, 2006), and auditory cortex lesion (Druga and Syka, 2001; Bowen et al., 2003; Rybalko et al., 2006; Clarkson et al., 2010a,b,c).

In the IC partial deafferentation of the ascending auditory pathway reduced sound-evoked inhibitory collicular responses (Semple and Kitzes, 1985; Bledsoe et al., 1995; McAlpine et al., 1997; Vale and Sanes, 2002). Whole cell patch recordings in brain slices showed that bilateral deafness induced a decrease in lateral lemniscus neurotransmitter release and synaptic strength variations in both excitatory and inhibitory IC synapses (Vale and Sanes, 2002). It is known that changes in the balance of excitation and inhibition trigger synaptic plasticity, adapting biophysical membrane properties (synaptic strength and axon conductance properties) or modifying the synthesis and trafficking of receptors (Perez-Otano and Ehlers, 2005; Turrigiano, 2012). Holt et al. (2005) suggested unbalanced excitation and inhibition as a basis for long-term (90 days) changes in adult IC gene expression after inactivation of the ascending auditory pathway by bilateral cochleotomy. Ascending lemniscal connections to the IC include both excitatory and inhibitory fibers (Riquelme et al., 2001). Therefore ascending deafferentation included a loss of both excitatory and inhibitory inputs. However, the descending pathway is solely excitatory, and its absence should lead to a strong unbalance of excitation and inhibition with consequences in gene expression, which are currently unknown.

We have previously used an auditory cortical ablation model in adult rats to analyze long-term plastic changes in the IC (Clarkson et al., 2010a,b,c). We demonstrated that restricted cortical ablation in acoustically stimulated animals strongly decreased c-Fos immunoreactivity in IC neurons at 15 days post-lesion, with a significant recovery taking place after 90 days (Clarkson et al., 2010a). It is well known that c-Fos immunoreactivity is an anatomical marker of neuronal activity (Bullitt, 1995), and that its alteration may be related to changes in neuronal transcription (Kovacs, 2008). Based on our c-Fos results (Clarkson et al., 2010a), we suggest that time-dependent activation of IC neurons after long-term cortical deprivation may be a consequence of the reorganization of auditory pathway projections. In the Central Nervous System, mechanisms of neural plasticity underlying long-term network reorganization such as collateral sprouting and pruning, cell death, neurotransmitter regulation, or glial functional differences, are reflected in changes in levels of gene expression (Wieloch and Nikolich, 2006). To gain insights into long-term plastic changes after descending IC deafferentation, we have used in our auditory cortex ablation animal model (Clarkson et al., 2010a,b,c) a Gene Chip Microarray technology, validated by quantitative reverse transcription real-time PCR (RT-qPCR).

## Materials and Methods

### Experimental animals

All experiments were performed according to national (R.D. 1201/2005) and EU regulations (DOCE L 222; 24-08-1999) for use and care of animals in research. Nine male rats (Wistar albino, Charles River Laboratories) weighing 230 g and 12 weeks of age at the beginning of experiments were used. Animals were free of ear infection and for a quick assessment of normal hearing we used in all cases bilateral finger friction test. For DNA microarrays analysis we used both IC (ipsilateral and contralateral) from three animals in each group (naïve control, 15 days post-lesion and 90 days post-lesion). For RT-qPCR three replicates from control and 90 days post-lesion group (both ICs) were randomly selected and run in triplicate twice for each gene product (25 genes).

### Surgery and auditory cortex lesion localization

Animals were anesthetized with ketamine chlorhydrate (30 mg/Kg, Imalgene^®^ 1000, Rhone Méreuse, Lyon, France) and xylazine chlorhydrate (5 mg/Kg Rompun^®^, Bayer, Leverkusen, Germany). Unilateral ablation by aspiration of the left auditory cortices (primary – Au1, dorsal – AuD, and ventral – AuV areas), including cortical layers V and VI, was carried out under stereotaxic control using a stereotaxic frame (David Kopf Ins., Tujinga, CA, USA) following a procedure described in detail elsewhere (Clarkson et al., 2010b).

Following the appropriate number of days post-lesion, ablated, and naïve control animals were deeply anesthetized with sodium pentobarbital (60 mg/kg) and decapitated. After quickly exposing the brain stem, both ICs were removed and the brain was stored overnight in a solution of 4% paraformaldehyde in phosphate buffer (PB) 0.1 M, pH 7.4. Finally, the brains were cryoprotected in 30% sucrose in 0.1% PB and serially sectioned at 40 μm to quantify the percentage of auditory cortices affected by ablation (Clarkson et al., 2010a,b).

### RNA isolation

Collected ICs were homogenized and total RNA was purified using TRIZOL^®^ (Gibco BRL, Gaithersburg, MD, USA). RNA quality was assessed and quantitated by Agilent 2100 Bioanalyzer software (Agilent Technologies, Palo Alto, CA, USA) associated with a RNA 6000 Nano kit. A RNA integrity number (RIN) >8.0 were found in all samples. In addition, further RNA purification using an RNeasy Mini Kit for RNA clean-up (Qiagen Sciences, Maryland, USA) was performed.

### Microarray, data, and ontological analysis

Microarray analyses were performed at the Cancer Research Center (Centro de Investigacion del Cancer – CIC) at the University of Salamanca (Spain). Labeling and hybridizations were performed according to Affymetrix protocols. One hundred to three hundred nanograms of total RNA were amplified and labeled using the WT Sense Target labeling and control reagents kit (Affymetrix Inc., Santa Clara, CA, USA), and hybridized to Rat Gene 1.0 ST Array (Affymetrix). Washes and scans were performed using GeneChip System of Affymetrix (GeneChip Hybridization Oven 640, GeneChip Fluidics Station 450, and GeneChip Scanner 7G).

Following image analysis, microarray data were imported into GeneSpring GX 7.3 (Agilent Technologies). RMA (Robust Multi-array Analysis), a method for normalizing and summarizing probe-level intensity measurements, was used. For this analysis, all genes that did not change between samples were excluded. We compared the experimental samples with naïve controls; all our samples passed a high data quality control, showing a high homogeneity intra-group (Datasheet 1 in Supplementary Material). Potential differential expression was determined with a one-way analysis of variance ANOVA (variances not assumed to be equal) and subsequently an unpaired *t*-test, *p* < 0.05, filtered for 1.5-fold was made in order to search differences in the gene expression (control samples were used as basal levels). Further processing including functional analysis and overrepresentation calculations based on Gene Ontology (GO) Annotation Tool and publication data from Database for Annotation, Visualization, and Integrated Discovery were made with GeneSpring GX 7.3 and Database for Annotation, Visualization, and Integrated Discovery (DAVID) Bioinformatics Resources 6.7 (http://david.abcc.ncifcrf.gov/; Dennis et al., 2003; Huang et al., 2009).

Only genes with a Fold Change (FC) >1.5 (up or down), were considered for analysis (Datasheet 1 in Supplementary Material). From this group of genes we centered our attention in categories reported altered in the IC after auditory cortical lesion or are related directly to post-lesional plasticity (genes shown in Figures 2–4).

### Quantitative reverse transcription real-time PCR

Total RNA (2 μg), primed with oligo-dT, was reverse-transcribed into cDNA at 37°C for 2 h using the first-strand cDNA synthesis kit (Promega Corporation, Madison, WI, USA). In all cases, a reverse transcriptase negative control was used for testing genomic DNA contamination. RT-qPCR was carried out on a real-time detection instrument (ABI Prism 7300 system) in 96-well optical plates using TaqMan Universal PCR Master Mix and Assay on Demand primers and probes. Probe sets used are listed in Datasheet 2 in Supplementary Material. Reaction components included: 2X TaqMan Universal Master Mix with UNG, 450 nM unlabeled PCR primers, 125 nM FAM dye-labeled TaqMan MGB probe, and 1 μL cDNA reaction product in a 10 μL total reaction volume. PCR conditions were as follows: 2 min at 50°C, 10 min at 95°C and 40 cycles of 15 s at 95°C and 1 min at 60°C. Relative quantities were calculated using the 2^−ΔΔCt^ analysis method (Schmittgen and Livak, 2008) with *GAPDH* (*Glyceraldehyde-3-phosphate dehydrogenase*: Rn99999916_s1) as the endogenous control. 2^−ΔΔCt^ values were analyzed using One-way analysis of variance (ANOVA; *p* < 0.05) with a *post hoc* Student Newman-Keuls test (*p* < 0.05) was performed for statistical analysis.

## Results

We analyzed differences in gene expression profiling in the IC in the short (15 days) and long-term range (90 days) after unilateral auditory cortex ablation. In addition, as this is an anatomically asymmetric projection in terms of innervation density, we also studied the differences in gene expression between the ipsi- and contralateral IC at each time post-lesion. Comparisons between control and deafferented groups showed gene products corresponding to a total of 24,070 probes (of the 27,342 total probes on the arrays) which were confidently detected based on signal intensity at a fixed value above background level (See Material and Methods, Robust Multi-array Analysis, RMA).

To enhance biological interpretation of the differentially expressed genes we performed function enrichment analysis for these genes using the functional classification tool “DAVID Bioinformatics.” Our results indicated that, although there were many over-represented biological function group of genes as shown in Datasheet 3 in Supplementary Material, the majority of them were related to a few functional categories. In our study the most relevant categories included genes related to neurotransmission and signal propagation such as receptors for glutamate, glycine, acetylcholine, or serotonin. This classification also involves enzymes and neurotransmitter transporters (glutamate, γ-aminobutyric acid (GABA), and glycine) and different types of channels (chloride, potassium, sodium, and calcium). Other functional categories notably detected include genes encoding proteins implicated in neural/synaptic plasticity, axonal growth/degeneration, myelin organization, and regulation, sprouting, neuroprotection, immune response, regulation of apoptosis, autophagy, and cell proliferation, migration, and differentiation.

**Figure 1:**
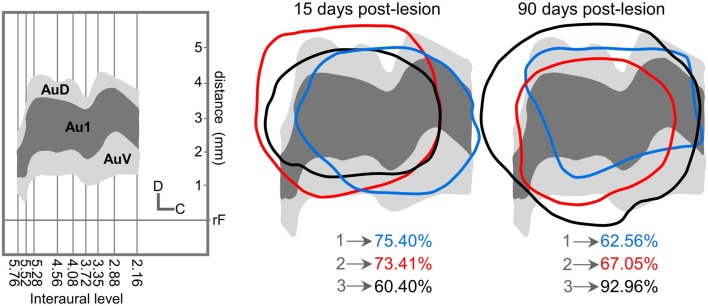
**Location and percentage of auditory cortical lesion for all cases used after 15 and 90 days post-lesion**. On the left, the diagram shows the representation in our cases of primary and secondary auditory cortices (Clarkson et al., 2010a) for all interaural levels drawn by Paxinos and Watson (2005). We used like reference for the coordinates of the vertical axis the distance in mm from rhinal fissure. Right side, superimposition of each lesion contours over auditory cortex diagram. AuC, Primary auditory cortex; AuV, Ventral auditory cortex; AuD, Dorsal auditory cortex.

All unilateral ablations were restricted in depth to the cortical gray matter (including layer 6) and their extension mainly affected the primary and secondary auditory cortices with an average of 69.74 + 4.7% for 15 days post-lesion group and 74.19 + 9.4% in the group 90 days post ablation (Figure 1).

### Control vs. 15 days post-lesion in the ipsilateral IC and control vs. 15 days post-lesion in the contralateral IC

**Figure 2:**
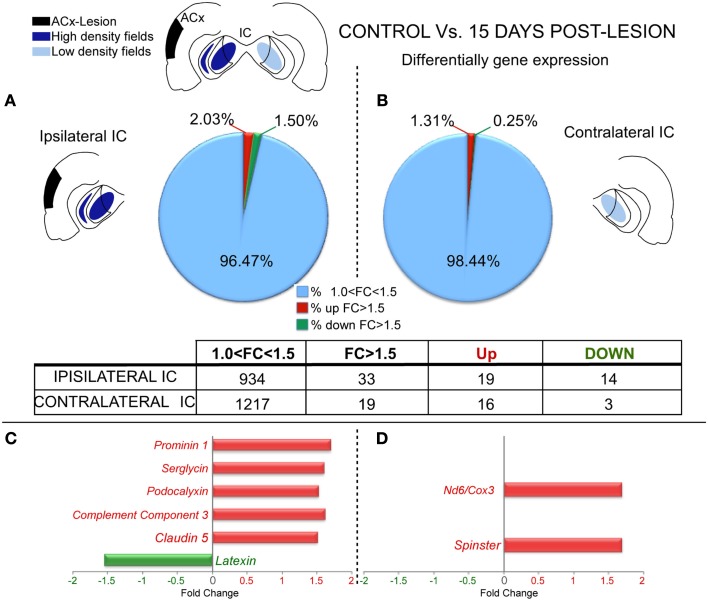
**Differential gene expression between control and lesioned cases in the inferior colliculus 15 days after ablation of the auditory cortex**. The insets show in black the unilateral auditory cortical lesion area and in blue the location of cortical projection fields in both IC. Notice that dark blue means a higher density of terminals and light blue a weaker cortical projection density. **(A,B)** Number of regulated genes in the IC ipsilateral **(A)** and contralateral **(B)** to the cortical lesion. The blue sector indicates the genes whose change was 1 < FC < 1.5. The red (up-regulation) and green (down-regulation) sectors indicate the percentage of genes whose change was greater than 1.5-fold. Notice for both ICs a predominant up-regulation in comparison to down-regulation of gene expression. **(C,D):** Bar graph showing functional analysis of the most representative genes for comparison between controls group and IC ipsilateral **(C)** and contralateral **(D)** IC to the cortical lesion. No changes *: Number of genes without significantly expression changes. Acx-Lesion, Auditory cortex lesion; IC, Inferior colliculus; *ND6/COX3*, *NADH dehydrogenase subunit 6 | cytochrome c oxidase subunit 3*.

Microarray comparisons between control and lesioned cases after 15 days showed that the genomic profile in the IC ipsilateral to the side of the lesion was slightly affected with 19 genes significantly up-regulated (FC > 1.5 and *p* < 0.05) and 14 genes significantly down-regulated (Figure 2A and Datasheet 1 in Supplementary Material). The IC contralateral to the side of the lesion also followed a similar trend with 16 genes up-regulated and three down-regulated (Figure 2B and Datasheet 1 in Supplementary Material).

After examining the potential function for each significantly affected gene per lesioned side we found that after 15 days post-lesion, the ipsilateral IC which had lost a denser cortical projection had a major up-regulation in several genes involved in inflammatory pathways (*Serglycin*, *Srgn; Podocalyxin*, *Podx; Complement Component 3*, *C3; and Claudin 5*, *Cldn5*) and stem cell regeneration (*Prominin 1*, *Prom1*). On the other hand, one gene directly related with neuroprotection (*Latexin*, *Lxn*) was clearly down-regulated (Figure 2C). Furthermore, the contralateral IC that had lost a weaker projection showed an up-regulation in genes involved in inflammation processes (*NADH dehydrogenase subunit 6 | Cytochrome c oxidase subunit 3, Nd6|Cox3)* and synaptic growth (*Spinster*, *Spns*; Figure 2D).

### Control vs. 90 days post-lesion in the ipsilateral IC and control vs. 90 days post-lesion in the contralateral IC

**Figure 3:**
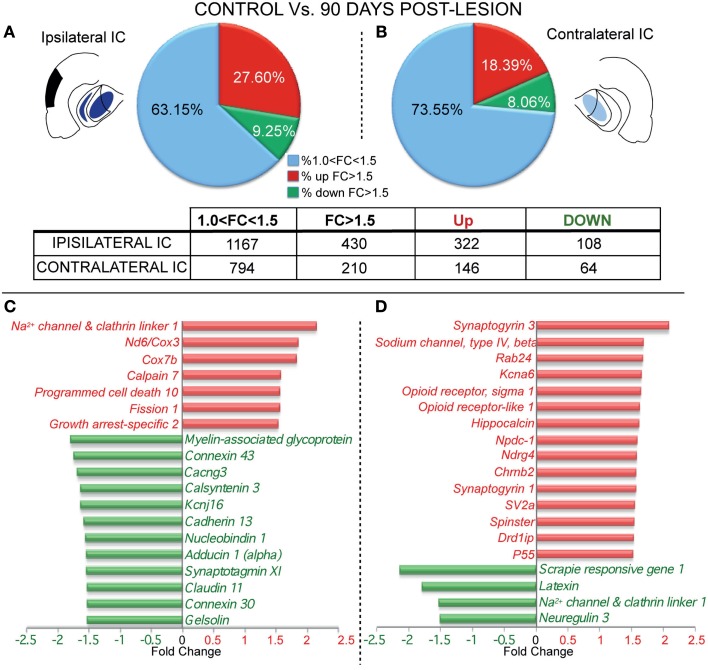
**Differential gene expression between control and lesioned animals in the inferior colliculus 90 days after ablation of the auditory cortex**. The insets show in black the unilateral auditory cortical lesion area and in blue the location of cortical projection fields in both IC. Notice that dark blue means a higher density of terminals and light blue a weaker cortical projection density. **(A,B)** Number of regulated genes in the IC ipsilateral **(A)** and contralateral **(B)** to the cortical lesion. The blue sector indicates the genes whose change was 1 < FC < 1.5. The red (up-regulation) and green (down-regulation) sectors indicate the percentage of genes whose change was greater than 1.5-fold. Bar graph shows functional analysis of the most representative genes for comparison between control group and ipsilateral **(C)** and contralateral IC **(D)** to the cortical lesion. ATPase-6, CO-III, NADH dehydrogenase subunit 6 | cytochrome c oxidase subunit 3; COX subunit VIIb, Cytochrome c oxidase subunit VIIb; KCNJ16, Potassium inwardly rectifying channel, subfamily J, member 16; RAB24, RAB24, member RAS oncogene family; Kcna6, potassium voltage gated channel, shaker related subfamily, member 6; NPDC1, neural proliferation, differentiation and control, 1; Nrdg4, N-myc downstream regulated gene 4; SV2a, synaptic vesicle glycoprotein 2a; Drd1ip, dopamine receptor D1 interacting protein.

The analysis of gene expression after 90 days post-lesion, on both the ipsi- and contralateral sides, showed greater changes compared to control than did the 15-days group. The ipsilateral side (90 days) displayed up-regulation of 322 genes and down-regulation in 108 genes (Figure 3A and Datasheet 1 in Supplementary Material). In the contralateral IC, 146 genes were up-regulated and 64 genes were down-regulated (Figure 3B and Datasheet 1 in Supplementary Material).

In particular, after 90 days post-lesion, gene function analysis showed that the ipsilateral IC had significant adjustments in genes involved in neurotransmission and signal propagation. Specifically, five were clearly down-regulated: *Calcium channel – voltage-dependent – γ3*, *Cacng3; Potassium inwardly rectifying channel*, *subfamily J*, *member 16*, *Kcnj16; Adducin 1 (alpha)*, *Add1; Claudin 11*, *Cldn11; and Calsyntenin 3*, *Clstn3*, and one was up-regulated (*Sodium channel and clathrin linker 1*, *Sclt1*; Figure 3C). We also found up-regulation in the expression of genes potentially involved in apoptotic processes (*Programmed cell death 10*, *Pdcd10; Growth arrest-specific 2*, *Gas2; Fission 1*, *Fis1; and Cytochrome c oxidase subunit VIIb*, *Cox7b*) and down-regulation in an anti-apoptotic gene (*Gelsolin*, *Gsn*). At the same time, *the NADH dehydrogenase*, *subunit 6 |cytochrome c oxidase III (Nd6|Cox3)* gene involved in inflammatory response also showed up-regulation at this time post-lesion. Additionally, genes involved in synaptic growth, such as *Myelin-associated glycoprotein* (*Mag*) *and Cadherin 13* (*Cdh13*), also displayed down-regulated expression, accompanied by up-regulation in one gene related to axonal degeneration (*Calpain 7*, *Capn7*). Furthermore, *Gap junction proteins α1 and β6 (Gja1 and Gjb6)*, also named *Connexin 43* and *30*, *respectively*, showed down-regulation in gene expression. Finally, relevant genes for calcium regulation like (*Nucleobindin 1*, *Nucb1*) and calcium sensors like *Synaptotagmin XI (Syt11)* were down-regulated (Figure 3C).

Ninety days after the cortical lesion, the contralateral IC exhibited larger changes in the expression of genes related with neurotransmission than at 15 days, showing an up-regulation in *Dopamine receptor D1 interacting protein gene* (*Drd1ip*, *also named Calcyon*)*; Synaptogyrin 3* (*Syngr3*)*; Synaptic vesicle related protein* (*SV2*)*; Opioid receptor-like 1* (*Oprl1*)*; Potassium voltage gated channel*, *shaker related subfamily*, *member 6* (*Kcna6*), *and Sodium channel*, *type IV*, *beta* gene (*Scn4b*; Figure 3D). Furthermore, *Neuregulin 3* (*Nrg3*) and *Sodium channel and clathrin linker 1 (Sclt1)* genes involved in neurotransmission were down-regulated. Interestingly, we found up-regulation in genes related to neural plasticity like *Opioid receptor*, *sigma 1* (*Oprs1*)*; Hippocalcin* (*Hpca*)*; P55; Synaptogyrin* family *(Syngr1 and 3)*, and also in genes that regulate synaptic growth, like *Spinster*, and cell proliferation, such as *N-myc downstream regulated gene 4*, (*Ndrg4*), and *Neural proliferation*, *differentiation and control*, *1* (*Npdc1*; Figure 3D). In contrast, we found at this time/side down-regulation in *Latexin*, which is involved in neuroprotection. Mixes of genes related with autophagy were also affected, with an up-regulation in *RAB24*, *member RAS oncogene family* and a down-regulation in *Scrapie responsive gene 1* (*Scrg1*; Figure 3D).

### Ipsi- vs. contralateral changes at 15 and 90 days post-lesion

Unbalanced cortical inputs to the IC induced by unilateral cortical ablation seem to induce complex gene regulation patterns which are better appreciated by comparing the IC ipsi – and contralateral to the ablation side. In naïve control group, we did a comparison between ipsilateral and contralateral IC profiling and no significant FC in gene expression were found. Moreover, both ICs ipsi- and contralateral to the cortical lesion showed quantitatively slight alterations in gene expression after short-term post-lesion (15 days), with changes in 14 genes (Datasheet 2 in Supplementary Material). Of these genes, two have a known function as mediators of inflammatory processes and exhibit a down-regulation in the contralateral IC.

**Figure 4:**
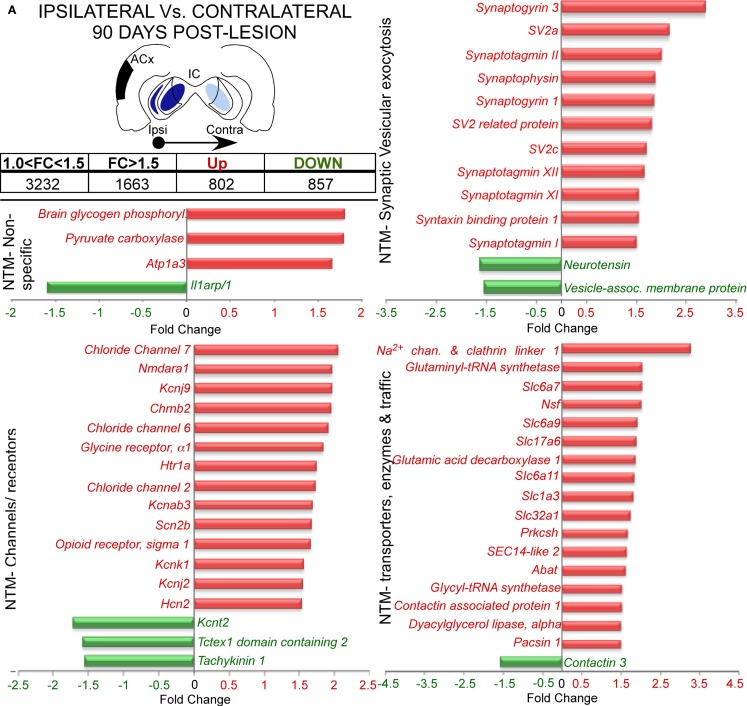

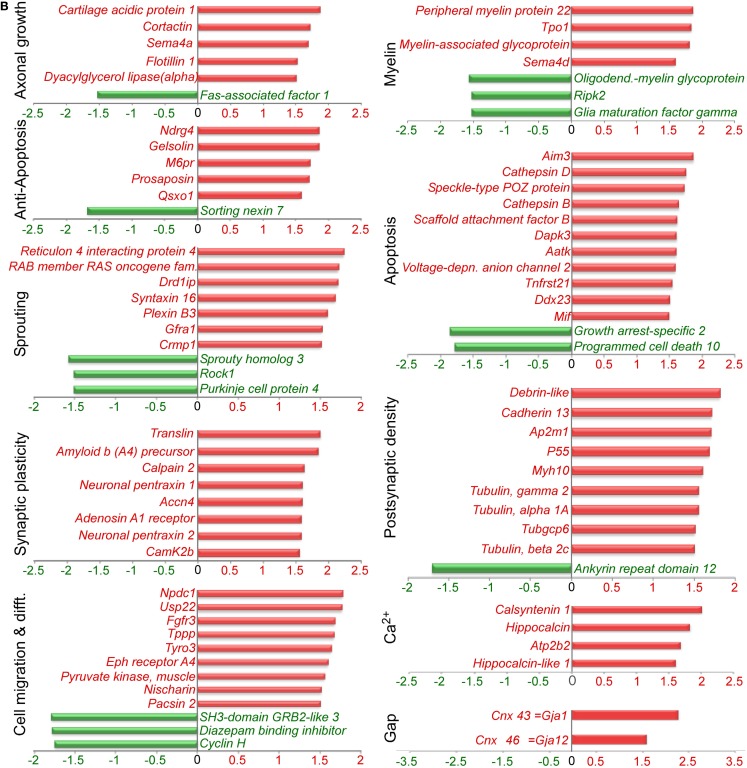
**Several of the most representative differentially expressed genes in the comparison between 90 days post-lesion in the ipsilateral IC vs. 90 days post-lesion in the contralateral IC**. The inset shows in black the unilateral auditory cortical lesion area and in blues the location of cortical projection fields in both inferior colliculus. Notice that dark blue means a higher density of terminals and light blue a weaker cortical projection density. Graph bar showing functional analysis of the most representative genes in each functional category. **(A)** Neurotransmission (NTS). **(B)** Axonal growth, anti-apoptosis, sprouting, synaptic plasticity, cell migration and differentiation, myelin, apoptosis, postsynaptic density, Ca^2+^ and Gap junctions.

At long-term post-lesion (90 days), a much larger adjustment in gene expression was found, with 1659 probes significantly altered, i.e., 802 genes up-regulated and 857 genes down-regulated (Figure 4A and Datasheet 1 in Supplementary Material). Analysis of genes function showed changes in 54 genes related with neurotransmission. These changes covered a wide range of neurotransmission processes, with up-regulation of glutamate receptor genes (*Glutamate receptor*, *ionotropic*, *N-methyl d-aspartate; Grin1*, *or Nmdar1*), glycine receptor (*Glycine receptor alpha 1*, *Glra1*), acetylcholine receptor subunits (*Cholinergic receptor*, *nicotinic*, *beta polypeptide 2; Chrnb2*), a serotonin receptor subunit (*5-hydroxytryptaine-serotonin receptor 1A*, *Htr1a*) and other receptor-like *Oprs1*. We also found up-regulation in five neurotransmitter synthesis enzyme genes [for glutamate: *Glutaminyl-tRNA synthetase* (*Qars*); for GABA: *Glutamic acid decarboxylase 1* (*Gad1*) and *4-aminobutyrate aminotransferase* (*Abat*); for glycine: *Glycyl-tRNA synthetase* (*Gars*); and for endocannabinoid mobilization through metabotropic glutamate receptors (mGluRs): *Dyacylglycerol lipase alpha* (*Dagla*)]. The neurotransmitter transporters also were affected. Two glutamate transporters genes were up-regulated *Solute* *carrier family 17 (sodium-dependent inorganic phosphate cotransporter) member 6*, *Slc17a6*, or also named *Vesicular glutamate transporter 2* (*VGlut2*); and *Solute carrier family 1 (Glial high affinity glutamate transporter) member 3*, *Slc1a3* or *G*LAST. Two GABA transporters (*Solute carrier family 6 – member 11 and family 32 – member 1*, *Slc6a11*, and *Slc32a1*, respectively), one glycine transporter [*Solute carrier family 6 member 9*, *Slc6a9*] and one proline transporter [*Solute carrier family 6 member 7*, *Slc6a7*] were also up-regulated. Up-regulation affected genes related with receptor trafficking as well, such as those for α-amino-3-hydroxy-5-methyl-4-isoxazolepropionic acid (AMPA) receptors: *N-ethylmaleimide sensitive fusion protein* (*Nsf*) and *Contactin associated protein 1* (*Cntnap1*). Also for acetylcholine receptors: *SEC14-like 2* and for sodium channel internalization with *Sodium channel and clathrin linker 1 (SLCT1)*. Moreover, the *Contactin 1* (*Cntn1*) gene that regulates traffic and synaptic content of AMPA receptors showed down-regulation after 90 days post-lesion (Figure 4A).

In addition, voltage-operated and other non-ligand gated ion channel genes were also up-regulated. These included three *chloride channel* genes (*Clcn2*, *6*, *and 7*), five potassium channels [*K*^+^ *inwardly rectifying channel subfamily J – members 2 and 9* (*Kcnj2 and 9*); *K*^+^ *voltage gated channel shaker related subfamily β3* (*Kcnab3*); *K*^+^ *channel subfamily K – member 1* (*Kcnk1*); and *Hyperpolarization activated cyclic nucleotide-gated K*^+^ *channel 2* (*Hcn2*)] and a sodium channel [*sodium channel voltage-gate*, *type IIβ* (*Scn2b*)]. Additionally, a channel for potassium [*K*^+^ *channel subfamily T – member 2* (*Kcnt2*)], another for calcium regulation (*Tctex1 domain containing 2*, *Tctex1d2)* and one receptor for substance P (*Tachykinin 1*, *Tac1*) were down-regulated (Figure 4A). It is important that the synaptic vesicular machinery was also affected, with an up-regulation of genes involved in neurotransmitter release, as well as calcium sensors for exocytosis, such as *Syngr1 and 3*, the *Synaptotagmin* family (*Syt1*, *2*, *11*, and *12*) and *SV* family (*SV2a* and *c*), and genes related with synaptic vesicle plasticity such as *Synaptophysin* and genes associated with synaptic vesicle docking and fusion (*Syntaxin binding protein 1*, *Stxbp1*, and *Syntaxin 16*, *Stx16*; Figure 4A).

Furthermore, as shown in Figure 4B, we found up- or down-regulation in genes involved in axonal growth and guidance, such as *cortactin* (*Cttn)* and *Dyacylglycerol lipase*, *alpha* (*Dagla*). At the same time, we detected changes in genes involved in myelin organization and regulation as *Peripheral myelin protein 22* (*Pmp22*) and *Developmentally regulated protein TPO1* (*Tpo1*), *Myelin-associated glycoprotein* (*Mag*), and *Oligodendrocyte-myelin glycoprotein* (*Omg*). We also found both types of regulation in genes involved in sprouting [*dopamine receptor D1 interacting protein* (*Drd1ip)*, *Sprouty homolog 3* (*Spry-3*), and *Rho-associated coiled-coil containing protein kinase 1* (*Rock1*)], in synaptic plasticity [*Amyloid beta (A4) precursor protein (App)*, *Neuronal pentraxin 2 (Nptx2)* and *Calcium/calmodulin-dependent protein kinase II*, *β Camk2b*] and in postsynaptic density reorganization (*p55*, and *Tubulins γ2*, *α1A*, *and β2C*). In addition, even after 90 days post-lesion, we still found regulations (up or down) in apoptotic genes, such as *Cathepsins B and D* (*Ctsb and Ctsd*), *Growth arrest-specific 2* (*Gas2*) and *Programmed cell death 10* (*Pdcd10*), but at the same time we found an increase in anti-apoptotic gene expression [*N-myc downstream regulated gene 4* (*Ndrg4)* and *Gelsolin* (*Gsn*)]. This regulation was accompanied by changes in genes involved in cell migration and differentiation [e.g., *Neural proliferation*, *differentiation and control*, *1 (Npdc1)* and *Diazepam binding inhibitor* (*Dbi*)] and glial connexins [*Gap junction membrane channel protein alpha 12* (*Gja12*), and *gap junction protein*, *alpha 1* (*Gja1* or *Cnx43*)].

### RT-qPCR analysis

RT-qPCR analysis was used to confirm a subset of gene expression changes observed in the microarray analysis. Genes chosen for RT-qPCR confirmation were mainly selected 90 days after cortical ablation, based on ontological categories with potential functional roles.

Differentially regulated genes confirmed by RT-qPCR belong to a majority of functional categories already described (neurotransmission, neural/synaptic plasticity, axonal growth/degeneration, myelin organization and regulation, sprouting, neuroprotection, regulation of apoptosis, and autophagy). Thus, as shown in Table 1, altered gene expression of the majority of genes, but not all, were confirmed by RT-qPCR.

**Table 1:** **Comparison of gene expression at 90 days after unilateral auditory cortex ablation: Microarray vs. RT-qPCR data**.

	Transcripts cluster ID	Gene description	Gene symbol	Control vs. 90dpl-Ipsi		Control vs. 90dpl-Contra		90dpl-ipsi vs. 90dpl-contra		Array PCR ID
				Microarray (1)	RT-qPCR (2)	Microarray (1)	RT-qPCR (2)	Microarray (1)	RT-qPCR (2)	
Neurotransmission: receptors, binding proteins, transports and metabolism, transport_channels	10812879	5-hydroxytryptamine (serotonin) receptor 1A	Htr1a	NS	NS	NS	NS	**1.746**	4,093 ± 1,23*	Rn00561409_s1
	10824517	Cholinergic receptor, nicotinic, beta polypeptide 2 (neuronal)	Chrnb2	NS	NS	NS	NS	**1.954**	NS	Rn00570733_m1
	10843400	Glutamate receptor, ionotropic, *N*-methyl d-aspartate 1	Grin1 = NMDAR1	NS	NS	NS	2,509 ± 0,45*	**1.663**	1,814 ± 0,33*	Rn01436038_m1
	10897146	Glutamate receptor, ionotropic, *N*-methyl d-aspartate-associated protein 1 (glutamate binding)	Grina	NS	NS	NS	NS	**1.967**	NS	Rn00596289_g1
	10836734	Glutamic acid decarboxylase 1	Gad1	NS	NS	NS	3,607 ± 0,45*	**1.871**	2,135 ± 0,27*	Rn00690300_m1
	10748118	*N*-ethylmaleimide sensitive fusion protein	Nsf	NS	1,537 ± 0,03*	NS	2,277 ± 0,10*	**2.028**	1,481 ± 0,01*	Rn00612444_m1
	10760290	Neuronal pentraxin 2	Nptx2	NS	−1,745 ± 0,07*	NS	NS	**1.584**	NS	Rn01756377_m1
	10909621	Sodium channel, type IV, beta	Scn4b	NS	NS	**1.683**	NS	NS	NS	Rn01418017_m1
	10821824	Solute carrier family 1 (glial high affinity glutamate transporter), member 3	Slc1a3 = Glast	NS	NS	NS	NS	**1.814**	NS	Rn00570130_m1
	10707325	Solute carrier family 17 (sodium-dependent inorganic phosphate cotransporter), member 6	Slc17a6 = VGlut2	NS	2,375 ± 0,29*	NS	4,270 ± 0,71*	**1.89**	1,798 ± 0,30*	Rn00584780_m1
	10841774	Solute carrier family 32 (GABA vesicular transporter), member 1	Slc32a1	NS	−1,570 ± 0,07*	NS	NS	**1.75**	1,524 ± 0,05*	Rn00824654_m1
	10857916	Solute carrier family 6 (neurotransmitter transporter, GABA), member 11	Slc6a11	NS	NS	NS	NS	**1.848**	1,871 ± 0,32*	Rn00577664_m1
Neurogenesis_neuronal differentiation	10752811	Amyloid beta (A4) precursor protein	App	NS	NS	NS	2,232 ± 0,30*	**1.848**	1,697 ± 0,23*	Rn00570673_m1
	10931154	diazepam binding inhibitor	Dbi	NS	NS	NS	1,543 ± 0,11*	**−1.778**	NS	Rn00821402_g1
	10834225	Neural proliferation, differentiation and control, 1	Npdc1	NS	NS	**1.585**	1,971 ± 0,16*	**1.773**	1,632 ± 0,13*	Rn01438701_g1
	10809100	*N*-myc downstream regulated gene 4	Ndrg4	NS	NS	**1.581**	2,371 ± 0,13*	**1.858**	1,347 ± 0,07*	Rn00582990_m1
Axonal growth_sprouty_axonal branching	10728676	Diacylglycerol lipase, alpha	Dagla	NS	−1,44 ± 0,10*	NS	NS	**1.509**	1,546 ± 0,15*	Rn01454304_m1
	10720813	Myelin-associated glycoprotein	Mag	NS	NS	NS	NS	**1.811**	NS	Rn00567868_m1
	10803158	Rho-associated coiled-coil containing protein kinase 1	Rock1	NS	NS	NS	NS	**−1.509**	NS	Rn00579490_m1
Synaptic vesicular exocytosis_calcium sensor	10902232	Synaptotagmin I	Syt1	NS	NS	NS	NS	**1.51**	NS	Rn00436862_m1
GLIA	10830189	Gap junction protein, alpha 1	Gja1 = Cnx43	**−1.749**	−1,842 ± 0,16*	NS	1,590 ± 0,05*	**1.593**	2,928 ± 0,09*	Rn01433957_m1
Cell death_ apoptosis_autophagy	10755728	Apoptosis-inducing factor, mitochondrion-associated 3	Aifm3	NS	NS	NS	3,588 ± 0,16*	**1.868**	2,367 ± 0,11*	Rn01405066_m1
	10894498	P55	P55	NS	NS	**1.52**	2,676 ± 0,34*	**1.684**	1,831 ± 0,24*	Rn01509468_g1
	10791500	Scrapie responsive gene 1	Scrg1	NS	NS	**−2.141**	NS	**−1.79**	NS	Rn00583743_m1
	10835757	Gelsolin	Gsn	**−1.527**	NS	NS	NS	**1.854**	2,033 ± 0,01*	Rn01438922_m1

*(1)Change in intensity, expressed as fold change*.

*(2)Expressed as fold change (2^−ΔΔCt^): mean ± SD*.

*NS, not statistically significant. Red indicates up-regulated; Green indicates down-regulated*.

*p < 0.05.

Microarray analysis identified many neurotransmission-related genes with significant increases in expression at 90 days after unilateral auditory cortex ablation (Figure 4A). From these genes, 12 were further assessed with RT-qPCR and showed comparable changes. A serotonin receptor (*Htr1a*), a glutamate receptor (*Grin1 or NMDAR1*), an enzyme for GABA synthesis (*Glutamic acid decarboxylase 1*, *Gad1*), a gene involved in the regulation of AMPA receptors (*N-ethylmaleimide sensitive fusion protein, Nsf*), a glutamate transporter *Slc17a6* (=*VGlut2*), and two GABA transporters (*Slc32a1* and *Slc6a11*) showed significant increases of 4.09-, 1.81-, 2.13-, 1.48-, 1.79-, 1.52-, and 1.87-fold at 90 days in the contra- (vs. ipsilateral) IC, respectively. Increased gene expression at the same time point was seen for a cholinergic receptor (*Chrnb2*), NMDA receptor associated protein (*Grina*), *Neuronal pentraxin 2 (Nptx2)*, and the glutamate transporter *Slc1a3* (=*GLAST*) using microarrays analysis, whereas RT-qPCR did not confirm such a significant increase. Conversely, *Nsf* and *VGlut2* had increased levels of expression at 90 days in the ipsilateral IC and genes like *Grin1* and *Gad1* also had increased levels of expression at the same time point in the contralateral IC by RT-qPCR analysis, whereas the microarray data did not show a significant change. Also, RT-qPCR for *Nptx2* and a GABA transporter (*Slc32a1*) showed a 1.74- and 1.57-fold decrease in expression at 90 days in the ipsilateral IC after unilateral auditory cortex ablation, whereas microarrays did not show significant changes. In contrast, increased gene expression was seen at 90 days in the contralateral IC for the *Sodium channel*, *type IV*, *beta* (*Scn4b*) using microarrays analysis, whereas the RT-qPCR did not show a significant increase.

Microarray results for the neurogenesis-related genes or neuronal differentiation, such as *Amyloid beta (A4) precursor protein* (*App*), *Neural proliferation*, *differentiation and control*, *1* (*Npdc1*), and *N-myc downstream regulated gene 4* (*Ndrg4)*, showed an increase in expression at 90 days in the contralateral IC vs. control and vs. ipsilateral IC, respectively, after unilateral auditory cortex ablation. On the other hand, decreased gene expression at 90 days in the contra vs. ipsilateral IC was seen in the *Diazepam binding inhibitor* (*Dbi*) using microarrays analysis, while the RT-qPCR did not show a significant increase. In contrast, this same gene showed 1.54-fold increase in expression at 90 days in the contralateral IC by RT-qPCR, whereas the microarray data did not show a significant change at this time point.

Other genes such as the enzyme involved in endocannabinoid mobilization *Dyacylglycerol lipase alpha* (*Dagla*), glial connexin *Gja1* (=*Cnx43*), *Apoptosis-inducing factor mitochondrion-associated 3* (*Aifm3*), *p55*, and *Gelsolin* (*Gsn*) assessed by RT-qPCR showed expression levels which were also increased at 90 days in the contra vs. ipsilateral IC, whereas *Myelin-associated glycoprotein* (*Mag*), *Rho-associated coiled-coil containing protein kinase 1 (Rock1)*, *Syt1*, and *Scrapie responsive gene 1* (*Scrg1*) did not show significant changes. Moreover, the gene encoding the gap junction protein *Cnx43* and *p55* showed 1.84-fold decrease and 2.68-fold increase in expression at 90 days post-lesion in the ipsilateral and contralateral IC (vs. control), respectively, after unilateral auditory cortex ablation. A number of the RT-qPCR confirmation analysis (*Dagla* at 90 days in the ipsilateral IC, and *Cnx43* and *Aifm3* at 90 days in the contralateral IC) which did not reach statistical significance, demonstrated expression profiles similar to those observed in the microarray. On the other hand, *Gsn* at 90 days in the ipsilateral IC and *Scrg1* at 90 days in the contralateral IC, showed changes in gene expression levels in microarrays, whereas the RT-qPCR did not show a significant increase.

As shown in Table 1, we performed RT-qPCR analysis on 24 genes at 90 days in the contra vs. ipsilateral IC after unilateral auditory cortex ablation and 15 of them showed similar changes in expression levels using microarrays analysis. Therefore, we used RMA for normalization that reflects more accurately the expression level of genes and a statistical method sufficiently stringent in assigning significance. We have observed differences in expression of some genes (e.g., *Nptx2*, *Slc1a3*, and *Scn4b*) using microarray analysis whereas RT-qPCR did not confirm such a significant variation. This situation was also described after microarray analysis in the IC by Holt et al. (2005), mainly in genes that are constitutively expressed at low levels. It should not be surprising that when constitute expression of genes is extremely low the threshold of microarrays are higher than qPCR arrays.

## Discussion

Unilateral auditory cortical deafferentation induces bilateral and asymmetric changes in gene expression profiling in the IC. In adults, this plastic response is time-dependent, with more extensive regulation at long-term post-lesion (90 days) than at short-term (15 days). Also, the nature of this reorganization is different for each time post-lesion, supporting the concept of a plastic ability in adults.

Auditory cortical ablation affects a large number of genes simultaneously; at 15 days post-lesion we found 52 genes (33 in the ipsilateral side and 19 in the contralateral side) whose expression changes. Meanwhile, 90 days after the lesion this regulation affects 640 genes in the IC (430 in the ipsilateral side and 210 in the contralateral side). Unilateral cortical ablation strongly affects the ipsilateral lesioned side at both times post-lesion (15 and 90 days), due to the loss of a denser innervation. Ipsilateral regulation was greater after long-term (90 days) than after short-term. This “late” regulation suggests an important role for adult neural plasticity events in this key auditory nucleus. After 15 days post-lesion, gene expression regulation was mostly related to an inflammatory response, whereas after 90 days regulation was linked mainly to sprouting phenomena and synaptic transmission. We already know that the auditory system in adults is able to induce gene modulation in the IC even after long periods of peripheral deafferentation (Holt et al., 2005). Our approach has focused on a comparatively minor contralateral IC deafferentation and a stronger ipsilateral IC deafferentation resulting from unilateral cortical ablation. Our results show that this misbalance is enough to induce a rearrangement in gene expression profiles in the IC, involving the pathways necessary to trigger neuronal plasticity in adults. Neurons convert environmental stimuli using a complex array of signaling pathways and transcriptional mechanisms to orchestrate long-lasting changes in their physiology through the synaptic activity-regulated transcription of new gene products (Lyons and West, 2011). After auditory cortical lesions, these long-term transcriptional profiling changes in the bilateral ICs are an indication of adult synaptic plasticity triggers, which are dependent on both post-lesioned time and density deafferentation.

Our results are a first look at the entire post-lesional auditory plasticity process. To assess an IC reorganization after cortical ablation both genomic and proteomic analysis are necessary. Genomic results cannot be extrapolated to protein production since it is known that there exist some mismatches between genomic and proteomic data in both brain cell culture (Howley et al., 2012) and most specifically in auditory system nuclei (Bush and Hyson, 2008; Wang et al., 2009a,b). Wang et al. (2009b) found 80 days after sound exposure that GlyR α subunit message was increased in the dorsal cochlear nucleus. However, the protein level was decreased. Mechanisms for this mismatch are still unknown, but studies demonstrate that information regarding post-translational modifications of the proteins and their translocation is not inherently encoded in the gene sequences and cannot be derived from mRNA expression (see Benoit et al., 2011 review).

### The ipsi- and contralateral IC 15 days after the lesion to the cortex

Functional analysis of IC gene profile expression 15 days after the lesion shows an up-regulation in*Serglycin*, a proteoglycan expressed primarily by immune cells (Kolset and Pejler, 2011) with a key role in inflammatory processes (Kolset and Tveit, 2008), suggesting that, in our model, cortical ablation induces in the short-term an inflammatory reaction in the ipsilateral IC which undergoes a larger loss of cortical descending afferents. In agreement with this finding, previous studies in the IC using a thiamine deficiency model to analyze inflammation, cellular stress, metabolism, and structural damage after focal neuronal death (Vemuganti et al., 2006) found an up-regulation in *Podocalyxin*, a cell adhesion regulator gene. More recently, other roles for this gene in neural development, neurite growth, branching, axonal fasciculation, and synapse formation after neuronal death or inflammation have been shown (Vitureira et al., 2010). In our experimental model, up-regulation of this gene in the ipsilateral IC suggests that this adjustment correlates with the density of the lesioned pathway. Furthermore, *Complement component 3* (*C3*), another inflammation-related gene, was found to be up-regulated in the IC in thiamin deficiency animals (Vemuganti et al., 2006) and in our ablated animals; supporting the idea of an ongoing inflammatory process at 15 days post-lesion which is stronger in the ipsilateral IC, accordingly with a heaviest preterminal fields degeneration after cortical ablation in the ipsilateral IC rather than the contralateral (Feliciano and Potashner, 1995). In addition, Stevens et al. (2007) have demonstrated that *C3* is a gene that tagged synapses to be eliminated during CNS development. These authors suggest that complement-mediated synapse elimination (synaptic stripping) may become aberrantly reactivated in neurological diseases. The process of synaptic elimination (pruning), even in adult animals, is important for rewiring neural circuits. Butz et al. (2009), using a model for analyzing cortical rewiring after deafferentation showed that even small changes in homeostatic equilibrium imply formation of new synapses or pruning of existing ones.

On the other hand, in the ipsilateral IC at 15 days post-lesion we also found down-regulated genes, like *Latexin* which is a carboxypeptidase inhibitor which mediates inflammatory responses (Aagaard et al., 2005), but it also known to be expressed by astrocytes, providing neuroprotective mechanisms (Yata et al., 2011). However, our results suggest that a typical astrocytic reaction does not take place in the IC, because we did not find any regulation in marker genes for reactive astrocytes like *Vimentin* or *Glial fibrillary acidic protein (GFAP)* which in the case of a thiamine deficiency model were up-regulated (Vemuganti et al., 2006).

Analyses of the contralateral IC 15 days after the cortical lesion show an up-regulation in the *Spinster* (*Spns*) gene, which is a negative synaptic growth regulator. The Drosophila *Spns* mutant shows a 200% increase in the number of synaptic endings and a deficit in presynaptic release (Sweeney and Davis, 2002). *Spinster* is also linked to a novel caspase-independent cell death pathway mediated by autophagy (Yanagisawa et al., 2003). These data speak in favor of a rearrangement process in the contralateral IC that has lost a weaker corticofugal projection compared with the side ipsilateral to the lesion. The focus of this regulation may be in synaptic pruning, looking for an efficient synaptic rewiring (Butz et al., 2009), or it may be related to changes in presynaptic release after lesion (Birthelmer et al., 2003), which affects activity in IC neurons (Nwabueze-Ogbo et al., 2002; Popelar et al., 2003).

In summary, microarray analyses at 15 days post-lesion show that genes mainly related to inflammatory processes were up-regulated in the ipsilateral IC. The relative increase in the activity of genes related to inflammation could be a consequence of extensive brain injury (Block et al., 2005). However the asymmetry of the corticofugal projection (Saldana et al., 1996; Bajo et al., 2007) geared toward the ipsilateral side, along with an ipsilateral predominance of changes in gene expression suggest a local consequence of terminal degeneration in the IC. The biological functions of genes, whose regulation is affected, indicate that beyond an inflammatory response an emergent plastic process, probably related with sprouting, and pruning on the adult collicular network, takes place bilaterally in the ICs after short-term post-lesion.

### The Ipsi- and contralateral IC 90 days after the lesion to the cortex

A much larger gene expression regulation pattern was found in the IC 90 days after the cortical lesion. Microarray comparisons between control and ipsilateral IC showed that changes affected remarkably genes involved in apoptosis. We found an up-regulation in *Programmed cell death 10* (*Pdcd10*) whose overexpression is sufficient to induce neuronal apoptosis (Lin et al., 2010). *Fission 1* (*Fis1*) which participates in apoptotic mitochondrial fission (Youle and Karbowski, 2005) was also up-regulated and so were *Growth arrest-specific 2* (*Gas2*) gene, a substrate of Caspase-3 (Sgorbissa et al., 1999), and *Cytochrome c oxidase subunit VIIb* (*Cox7b*) which is one of the last subunits that join the assembling cytochrome oxidase complex inducing apoptosis and affecting mitochondrial integrity (Fornuskova et al., 2010). We also found an up-regulation in *Calpain 7 (Capn7)*, which is associated after brain injury with neuron death and axonal degeneration (Saatman et al., 2010). In addition, we detected down-regulation of the *Calsyntenin 3* (*Clstn3*), a gene which overexpression accelerates neuronal death (Uchida et al., 2011). All this strongly suggests an ongoing cell death process in the ipsilateral IC at 90 days post-lesion. However we did not find changes in the expression of initiator or effector caspases genes triggering the apoptotic process. This could be related with limited microarray sensitivity. On the other hand, deprivation of auditory nerve input in adult animals does not result in significant neuronal loss in the cochlear nuclei (Harris et al., 2005), supporting the idea that in the auditory system mature neurons are less sensitive to apoptotic cell death. It could be that the regulation of apoptotic cascade genes observed by us may be related to other cellular pathways in which all these genes also performed a key role. Further specific proteomic experiments of caspases cell cascades will be needed to elucidate this problem, mainly because the regulation of apoptotic cascades is complex and involves transcriptional control as well as posttranscriptional protein modifications (Culmsee and Landshamer, 2006).

We also found in the ipsilateral IC 90 days after the lesion down-regulation of *Myelin-associated glycoprotein* (*Mag*), a gene able to inhibit axon regeneration after injury (Yiu and He, 2003). Studies *in vivo* demonstrate a modest but significant enhancement of axon regeneration in mice lacking *Mag* (Schnaar and Lopez, 2009). Down-regulation of this gene in our material could be an indication of axon elongation and indirectly of a sprouting process subsequent to lesion in the ipsilateral IC. However, at this same post-lesion time an oligodendrocyte-specific protein, *Claudin 11* (*Cldn11*), which encodes a molecular component present in tight junctions and which is also is involved in axon myelination, was down-regulated. This result probably indicates an inefficient axon myelination during axon elongation. *Cldn11* down-regulation may affect biophysical properties of myelinated axons in the IC, because it is known that *Cldn11*-null mice present a 60% decrease in conduction velocity (Devaux and Gow, 2008). A low effective compartmentalization of the myelin sheet of sprouting axon collaterals may be in accordance with previous results in which after 90 days of cortical lesion, sound stimulation-induced c-Fos immunoreactivity in the IC was only partially recovered (Clarkson et al., 2010a). In correspondence with this low activity in the IC at this survival time after the cortical lesion, genes related with synaptic activity also were affected with down-regulation in *Synaptotagmin-11* (*Syt11*, a Ca^2+^-sensor during vesicular trafficking, Inoue et al., 2007), *Calcium channel – voltage-dependent – γ3* (*Cacng3*, a gene with modulatory effects on the electrophysiological characteristic of the Ca_V_2.1 channel, Rousset et al., 2001) and also in *Potassium inwardly rectifying channel*, *subfamily J*, *member 16 (Kcnj16)* which plays a physiological role in the potassium buffering-action of brain astrocytes (Hibino et al., 2004). Holt et al. (2005) showed by RT-qPCR a rearrangement in synaptic transmission genes in the IC 90 days after cochlear ablation, with regulation affecting several glutamate and GABA receptors genes, supporting the hypothesis that, after long-term post-lesion, the adult auditory system is able to modify the gene neurotransmission machinery in the IC even after lack of auditory activity. Glial related genes such as *Connexin 30* and *43*, which are specific gap junction proteins that mediate cell-to-cell communication (Gemel et al., 2008) and *Cadherin 13* involved in regulation of cell growth, survival, and astrocyte proliferation (Huang et al., 2003) were down-regulated. Recently studies from connexin 30 and 43 show that these gap junctions mediate astroglial networks scale synaptic activity, as they define the concentrations and dynamics of extracellular ions and neurotransmitters during synaptic activity (Pannasch et al., 2011). These glia gene regulations after ablation are important for tripartite synapses model, in which brain function actually arises from the coordinated activity of a network comprising both neurons and glia (Perea et al., 2009).

The idea that in the ipsilateral IC 90 days after cortical ablation plastic or adaptive reorganizations have been switched on is also reinforced by previous data showing that sound-induced c-Fos immunoreactivity progressively increases from 90 to 180 days after cortical ablation (Clarkson et al., 2010a), showing that neuronal activity is still on its way to recovery.

After 90 days of the lesion, the contralateral IC showed up-regulation in genes involved in neurotransmission/signal propagation/synaptic plasticity such as *Sodium channel*, *type IV*, *beta* (*Scn4b*) whose overexpression induces neurite outgrowth, causes thickening of dendrites and increases the post synaptic density of neuronal spines (Oyama et al., 2006). Also *Potassium voltage channel*, *shaker related subfamily*, *member 6* (*Kcna6*) was up-regulated. This potassium channel regulates miniature inhibitory postsynaptic currents (mIPSCs) by regulating glycine release from the endings (Shoudai et al., 2007). In astrocytes *Kcna6* underlies part of the delayed rectifier potassium current (Smart et al., 1997). Another family of potassium channels like KCNQ5 and KCNK15 has been localized to neurons in the IC and are modulated by hearing loss (Caminos et al., 2007; Dong et al., 2009). Furthermore, up-regulation in *opioid receptor*, *sigma* *1* (*Oprs1*), found in our results, may be related to its role in neuronal plasticity, enhancing growth factor-induced neurite outgrowth (Hashimoto, 2010; Ruscher et al., 2011), and also regulating both Ca^2+^ entry and Ca^2+^ mobilization from endoplasmic reticulum stores (Monnet, 2005). Another opioid receptor involved in nociception, *Opioid receptor-like 1* (*Oprl1*) was also up-regulated, and is able to decrease acetylcholine release and dopamine release and elevates extracellular glutamate and GABA levels (Schlicker and Morari, 2000). All these changes were accompanied by an up-regulation in genes for neurotransmitter delivery at synaptic terminals. For example, in our material up-regulation of SV2a which exerts a main role in neurotransmitter uptake, vesicle targeting, and membrane fusion (Elferink and Scheller, 1995), as well as Synaptogyrin 1 and 3 (Syngr1 and 3), both of which play an essential function in synaptic plasticity without being required for neurotransmitter release (Belizaire et al., 2004). These data agrees with the idea that in adult animals the contralateral IC that loses the weaker projection from the cortex is still able, after long-term post-lesion, to enhance its genetic machinery to try to compensate the imbalance induced by lost cortical connections. This compensation could be the result of fine plastic modulations, which may include sprouting, pruning, and neurotransmission rearrangement. According to this idea, we also found an increase in *Hippocalcin* (*Hpca*) which encodes a calcium-binding protein considered relevant for synaptic plasticity, acting as a molecular link between Ca^2+^ entry through NMDA receptor and the subsequent endocytosis of AMPA receptor subunits in long-term depression (LTD; Palmer et al., 2005). Also it was shown that *Hpca* overexpression dramatically elongated neurites (Oh et al., 2008). Similarly, *Dopamine receptor D1 interacting protein* (*Drd1ip*) a known brain plasticity gene (Kruusmagi et al., 2007) that plays a specialized role in removal of synaptic AMPA receptor (Davidson et al., 2009) was up-regulated. So it was *N-myc downstream regulate gene 4* (*Ndrg4*) a contributor to neuronal differentiation, neurite formation, cell progression, and survival (Takahashi et al., 2005; Schilling et al., 2009), *Neural proliferation*, *differentiation, and control 1* (*Npdc1*) gene, which is able to down-regulate the proliferation of neural precursors (Dupont et al., 1997), and *Spinster* that is a negative regulator of synaptic regrowth (Sweeney and Davis, 2002) were also up-regulated. This balance between regrowth and growth suppression gene expression seems to be important to demonstrate rewiring network processes in the contralateral IC 90 days after lesion through activation of mechanisms involve in neuronal regulation.

In summary, up-regulation in genes related to axonal growth, receptor expression, and trafficking as well in channels and synaptic machinery may be compatible with plastic reorganization that is more active in the contralateral IC. These differences in the timing for plasticity activation between both sides may be explained by the asymmetrical loss of excitation due to the asymmetrical density of the descending connections from the cortex.

### Comparing ipsi- and contralateral changes at 15 and 90 days post-lesion

Comparison between ipsi- and contralateral IC after 15 days shows a small number of altered genes (14), contrasting with the larger number of regulated genes in the comparison (ipsi- vs. contralateral) after 90 days subsequent to the cortical lesion (1659 genes). Gene expression analysis between ipsi- and contralateral IC after 90 days following the cortical lesion showed up-regulation in potassium and chloride channels, and cholinergic, serotoninergic, glycinergic receptors in the contralateral IC. Biological function of up-regulated genes speaks in favor of a more active synaptic plasticity in the contralateral IC after long-term post-lesion. In particular, the NMDA receptor subunit coded by *Grin1* (*glutamate receptor*, *ionotropic*, *N-methyl d-aspartate 1*) and its associated protein coded by *Grina* (*glutamate receptor*, *ionotropic*, *N-methyl d-aspartate-associated protein 1*) were up-regulated in the contralateral IC. NMDA receptors are important for activity-dependent synaptic plasticity (Kalev-Zylinska et al., 2009; Rebola et al., 2010). *In vitro* experiments demonstrated that activation of NMDA receptors mediated a dramatic increase of both *c-fos* expression and intracellular calcium (Lerea et al., 1992). In a previous work both Calretinin (an indirect marker of Ca^2+^ influx- Clarkson et al., 2010b) and c-Fos in the IC contralateral to the lesion (Clarkson et al., 2010a) showed stronger immunoreactivity relative to the ipsilateral side. Future experiments will be need to demonstrate in our *in vivo* model a direct correlation among NMDA receptor activation, c-Fos activation, and changes in the intracellular Ca^2+^ concentration.

An extensive group of genes potentially involved in neurotransmitter release, such as four members of the *Synaptotagmin* family (*Syt1*, *2*, *11*, and *12*; Chapman, 2008; Rizo and Rosenmund, 2008; Sudhof and Rothman, 2009), were up-regulated in the contralateral side. In addition, genes involved both in expression and trafficking of *Synaptotagmin* family members, such as the *SV2* family (*SV2a*, *SV2c*, and *SV2 related protein*; Nowack et al., 2010) were also up-regulated in the contralateral IC. Not only were receptors and synaptic vesicle fusion genes up-regulated in the contralateral side after long-term lesion, but also this rearrangement was accompanied by an up-regulation in key enzymes for GABA (*4-aminobutyrate aminotransferase*, *Abat*, and *Glutamic acid decarboxylase 1*, *Gad1*), glycine (*Glycyl-tRNA synthetase*, *Gars*), glutamate (*Glutaminyl-tRNA synthetase*, *Qars*) and endocannabinoid synthesis (*Dyacylglycerol lipase alpha*, *Dagla*), all of them key components in short and long-term plastic synaptic changes. In addition, neurotransmitter transporters were up-regulated in the contralateral IC after long-term cortical ablation (for neuronal glutamate: *Vesicular glutamate transporter 2*, *VGlut2*, or *solute carrier family 17 member 6*, *Slc17a6*; for proline: *Slc6a7*; for glycine: *Slc6a9*; for GABA: *Slc6a11* and *Slc32a1;* and for glutamate: *Slc1a3* or *GLAST*). Up-regulation of both enzymes and transporter genes clearly suggests a post-lesion shift in the synthesis and recycling of neurotransmitters. These findings support the idea of a better recovery of neurotransmission in the contralateral IC probably due to the comparatively lower loss of excitation in that side after cortical lesion. All these data also suggest activity-dependent compensatory mechanisms in the contralateral IC, counteracting the most affected ipsilateral IC. In summary, these changes in gene expression are important due to the claim that regulation depends on the extent of deafferentation, and even in adult animals it is larger after long-term post-lesion with specific changes aimed at recovering activity loss in IC neurons.

## Author Contribution

Cheryl Clarkson Performed experiments, prepared figures and wrote the manuscript. M. Javier Herrero-Turrión. Performed RT-qPCRs experiments, analyzed statistically microarray data and drafted the manuscript. Miguel A. Merchán. Designed, coordinated, and supervised the study. All authors read and agreed the paper content.

## Conflict of Interest Statement

The authors declare that the research was conducted in the absence of any commercial or financial relationships that could be construed as a potential conflict of interest.

## Supplementary Material

The Supplementary Material for this article can be found online at http://www.frontiersin.org/Neural_Circuits/10.3389/fncir.2012.00086/abstract

Supplementary Datasheet 1 **Naïve control vs. 15 days and 90 days post-lesion**.

Supplementary Datasheet 2 **Primers PCR arrays**.

Supplementary Datasheet 3 **Funtional annotation of diferentially expresed genes**.

## Figures and Tables

**Figure A1:**
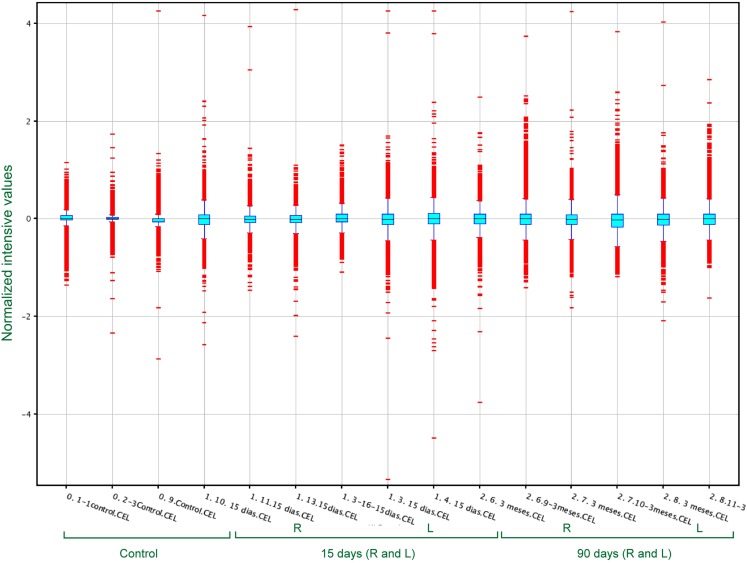
**Sample quality control**. Box whisker diagram showing the data distribution of each of the samples. The diagram shows the dispersion of each of the samples [control, 15 and 90 days post-lesion; inferior colliculus right (R) and left (L)] and the degree of similarity between replicas.
